# Successful Management of Invasive Pulmonary Nocardiosis and Aspergillosis in a Patient with T-Cell Lymphoma: A Case Report

**DOI:** 10.4137/ccrep.s817

**Published:** 2008-05-27

**Authors:** Khalid A. Al-Anazi, Mahmoud D. Aljurf, Fahad I. Al-Mohareb, Laila Al-Dabal, Mohammed Zaitoni, Majed Halim

**Affiliations:** 1 Section of Adult Hematology and Hematopoietic Stem Cell Transplant, King Faisal Cancer Centre, King Faisal Specialist Hospital and Research Centre, P.O. Box: 3354, Riyadh 11211, Saudi Arabia.; 2 Section of Pulmonary Medicine, Department of Medicine, King Faisal Specialist Hospital and Research Centre, P.O. Box: 3354, Riyadh 11211, Saudi Arabia.; 3 Section of Infectious Diseases, Department of Medicine, King Faisal Specialist Hospital and Research Centre, P.O. Box: 3354, Riyadh 11211, Saudi Arabia.

**Keywords:** angioimmunoblastic T-cell lymphoma, invasive pulmonary infections, *Aspergillus niger*, *Nocardia asteroides*

## Abstract

In patients with malignant hematological disorders receiving immunosuppressive therapy, invasive pulmonary infections are serious complications that are associated with high morbidity and mortality. In immunocompromised hosts with impaired cellular immunity, two or more organisms may coexist leading to a wide range of clinical and radiological manifestations.

Reported here is an old man who was diagnosed to have angioimmunoblastic T-cell lymphoma at King Faisal Specialist Hospital and Research Centre in Riyadh in December 2004. The lymphoma was treated with various immunosuppressive agents including alemtuzumab. In October 2006, the patient was admitted with severe bronchopneumonia caused by *Nocardia asteroides* and *Aspergillus niger* that was complicated by septic shock. The invasive pulmonary infections were successfully treated with trimethoprim-sulphamethoxazole, amikacin and liposomal amphotericin-B (amBisome).

## Introduction

Infections are the most common cause of morbidity and mortality in patients with malignant disorders (Neuburger and Maschmeyer, 2006). Despite the availability of various antimicrobial agents, pneumonia constitutes the sixth cause of death and the number one cause of death from infection (Waite et al. 2006). Pneumonia can be particularly life-threatening in the elderly, in patients with pre-existing pulmonary or cardiac conditions and in immunocompromised individuals (Waite et al. 2006; Rolston, 2001).

The spectrum of pulmonary infections depends upon the underlying immunological abnormalities (Rolston, 2001). In cancer patients, several immunological defects may be present, thus making them susceptible to a wide range of opportunistic infections (Rolston, 2001). In individuals with impaired cellular immunity, viral infections e.g. cytomegalovirus (CMV) predominate and may coexist with bacterial (e.g. Legionella, Nocardia etc), mycobacterial and fungal (e.g. Aspergillus, Histoplasma etc) infections (Rolston, 2001). However, pulmonary Nocardia and Aspergillus coinfections have been reported in hematopoietic stem cell transplant recipients and in patients with glomerulonephritis treated with corticosteroids (Hamadani et al. 2008; Varma et al. 1993).

## Case Report

**Figure 1:**
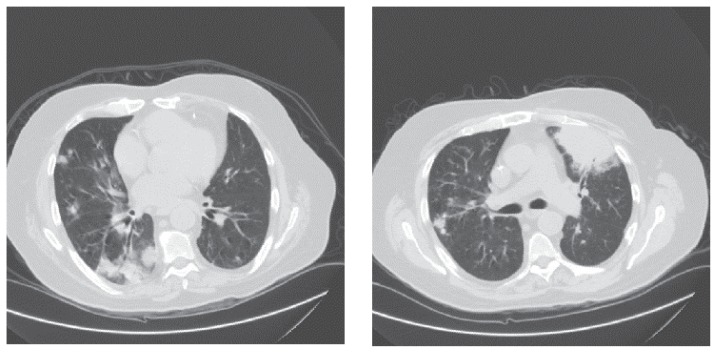
A high resolution CAT scan of the lungs. The CAT scan shows: bronchiectatic changes, areas of pneumonic consolidation and diffuse nodular pulmonary infiltration.

A 67 years old male, a non-smoker, with history of ischaemic heart disease, status post—coronary artery bypass graft, and bronchiectasis was diagnosed to have angioimmunoblastic T-cell lymphoma (AIBTCL), stage III B at King Faisal Specialist Hospital and Research Centre (KFSH&RC) in Riyadh in late December 2004. He presented with: fever, skin rash, generalized external lymphadenopathy and splenomegaly. The blood counts, blood film and bone marrow biopsy were all normal. The renal and hepatic profiles as well as the immunoglobulin levels were within normal limits. Serological tests for CMV, Epstein-Barr virus (EBV), hepatitis C virus and HIV were negative. Computed tomography (CT) scans of chest, abdomen and pelvis showed numerous small lymph nodes in mediastinal, mesenteric and retroperitoneal areas in addition to splenomegaly with focal splenic lesions. Skin biopsy showed atypical lymphohistiocytic changes. A right axillary lymph node biopsy revealed diffuse infiltration with: immunoblasts, lymphocytes, polymorphs, plasma cells, histiocytes and eosinophils with considerable proliferation of small blood vessels. The immunohistochemical stains showed positive: CD1A, CD3, CD4, CD7, CD8, CD15, CD20, CD21, CD30, CD45, CD68 and S100. The gene rearrangement studies revealed a monoclonal T-cell population. The AIBTCL was treated with: prednisone, mycophenolate mofetil and subcutaneous alemtuzumab: 30 mg/month for 4 months. Thereafter the patient developed: CMV infection treated with IV ganciclovir and pulmonary embolism treated with IV heparin then oral warfarin. On 29/10/06, the patient was readmitted with two week history of: fever, cough productive of yellowish sputum and mild dyspnea. He denied any associated chest or abdominal pain, headache or bleeding from any site. Physical examination revealed an unwell elderly male who was in mild respiratory distress. The temperature was 38.7 °C, blood pressure (BP): 112/68 mmHg, pulse rate: 92/minute, respiratory rate: 20/minute and oxygen saturation was 93% on room air. There was pallor but no cyanosis, leg oedema, jaundice or external lymphadenopathy. The inspiratory volume was decreased and coarse crackles were heard over mid and lower lung fields bilaterally. There was no abdominal distension, tenderness or palpable organomegaly. Cardiovascular and neurological examinations did not reveal any abnormality. Full blood count (FBC) showed: WBC: 1.83 × 10^9^/L, Hb: 109 g/L and PLT: 315 × 10^9^/L. Differential cell count (DCC) showed neutrophils of 1.1 and lymphocytes of 0.3. Renal and hepatic profiles were all within normal limits. Blood, urine, stool cultures and CMV antigen test were all negative. Chest radiography showed bronchiectatic changes involving both lower lobes and bilateral nodular infiltration consistent with severe pneumonia. The patient was commenced on IV tazobactam-piperacillin: 4.5 grams thrice daily and gentamicin 2 mg/kg IV twice daily in addition to oxygen via mask at 2–4 Liter/minute and IV fluids at rate of 50–80 cc/hour. Initially the patient had partial response then he started to have higher pyrexia and respiratory distress. On 6/11/2006, the patient was experiencing high grade fever and rigors and his BP dropped to 85/45 mmHg. Septic screens were repeated and the IV antibiotics were replaced by IV meropenem 1 gram thrice daily and IV vancomycin 1 gram twice daily. Later on, BP improved and fever started to subside. A repeat CT scan of the chest showed: bronchiectatic cavities involving the lower lobes of both lungs, bilateral nodular infiltration, areas of segmental consolidation and bilateral pleural thickening (Fig. 1). Echocardiogram showed no vegetations, pericardial effusions or valvular defects and brain CT scan showed no evidence of space occupying lesions. Bronchoscopy was performed and BAL fluid showed scattered lipid laden macrophages and gram positive rods identified later on as *Nocardia asteroides* (*N. asteroides*). Special stains for fungi were positive and the fungus was identified as *Aspergillus niger* (*A. niger*). BAL fluid was negative for acid fast bacilli, pneumocystis carinii, viral cytopathy and malignant cells. Meanwhile, the previously taken sputum cultures grew branching gram positive rods identified as *N. asteroides*. However, sputum cultures were negative for acid fast bacilli, candida and aspergillus. Aspergillus galactomannan test was positive. Consequently, the following management modifications were made: vancomycin was stopped and meropenem was continued, IV liposomal amphotericin-B (amBisome) 5 mg/kg/day was commenced in addition to oral trimethoprim-sulphamethoxazole (TMP/SMZ) 960 mg thrice daily as well as IV amikacin 15 mg/kg/day. Later on, the patient started to improve clinically and radiologically and oxygen requirements decreased gradually. One week later, the patient sustained his hemodynamic stability and his clinical improvement so IV meropenem was discontinued. On 20/11/2006, IV amikacin was stopped and IV amBisome was replaced by oral voriconazole. Two days later; the patient was totally asymptomatic and his physical examination showed few basal crackles with good air entry bilaterally. FBC showed WBC: 2.25 × 10^9^/L with neutrophils of 1.4, Hb: 94 g/L and PLT: 147 × 10^9^/L. Renal and hepatic profiles were normal. He was discharged on: voriconazole 200 mg orally twice daily and TMP/SMZ 960 mg orally thrice daily for 6 weeks in addition to warfarin, omeprazole, prophylactic valganciclovir as well as ventolin and atrovent inhalers. Thereafter, the patient had regular follow up at the hematology outpatient clinic and he remained clinically stable.

## Discussion

AIBTCL, an intermediate grade/aggressive mature peripheral T-cell lymphoma, was first described in the 1970s as a distinct clinical syndrome characterized by: pruritic skin rash, generalized lymphadenopathy, hepatosplenomegaly, hypergammaglobulinemia, anemia and constitutional B symptoms. It occurs in elderly individuals and has no known etiology, although it has associations with: various infections (e.g. EBV, CMV, hepatitis C virus, human herpes viruses 6 and 8, tuberculosis and cryptococcus), antibiotics and autoimmune disorders (e.g. rheumatoid arthritis, vasculitis and thyroid disease) (Dogan et al. 2003). Lymph node histology shows distinctive partial effacement of normal architecture by polymorphic inflammatory infiltrates including large blasts and marked vascular proliferation. The monoclonal T-cell population expressing CD3 and CD4 and the multiple clonal cytogenetic abnormalities are the other distinguishing features. Unfortunately there is no curative treatment, however, single agents such as corticosteroids, cyclosporine-A, alpha-interferon, thalidomide, fludarabine and 2-chlorodeoxyadenosine as well as combinations of cytotoxic drugs e.g. CHOP regimen (cyclophosphamide, doxorubicin, vincristine and prednisone) have been employed in the treatment of AIBTCL. The clinical outcome is generally poor and most of the deaths are infection related (Dogan et al. 2003).

Expression of CD52 occurs in 40% of patients with peripheral T-cell lymphoma. Although other factors may play a role in the in vivo response to alemtuzumab (Campath 1H), an anti-CD52 monoclonal antibody, the estimation of CD52 expression may provide a rationale for the selection of patients with a higher probability of treatment response (Pacciluga et al. 2007). The feasible chemoimmunotherapy (CHOP and alemtuzumab) combination has been shown to be an effective therapeutic modality for peripheral T-cell lymphoma, producing high rates of complete remission, but has also been associated with hematologic toxicity e.g. severe neutropenia and infectious complications including reacivation of CMV and Jacob-Creutzfeldt virus, invasive pulmonary aspergillosis (IPA) in addition to bacterial sepsis and pneumonia (Gallamini et al. 2007).

All neutropenic patients with pyrexia of unknown etiology having normal chest roentgenograms should undergo high resolution CT scanning as these scans have been shown to have 87% sensitivity and 57% specificity in detecting pneumonic infiltrations in febrile neutropenic patients with unknown foci of infection (Heussel et al. 1999). The favorable safety record, the good diagnotic yield and the frequent therapeutic implications strongly support the use of BAL for the evaluation of pulmonary infiltrates in neutropenic immunocompromised patients. BAL should be combined with the analysis of several sputum cultures as the combined diagnostic yield may reach 63% (Peikert et al. 2005). Current management strategies for febrile neutropenic patients emphasize on risk assessment (Picazo, 2005). The development of risk stratification models has allowed the identification of low risk patients who can exclusively be treated with oral antimicrbials as outpatient (Sipsas et al. 2005). The most important determinants of infection risk are the severity and the duration of neutropenia (Picazo, 2005). Monotherapy with the newer broad spectrum antimicrobials has tended to replace the classic combination therapy. Prompt initiation of empirical antimicrobial treatment has remained the gold standard. However, empirical administration of glycopeptides, e.g. vancomycin, without documentation of gram-positive infection is no longer favored. The initiation of empirical antifungal therapy in persistently febrile neutropenic patients has become a common practice, especially recently, after the introduction of the new, more effective and less toxic antifungal agents (Sipsas et al. 2005).

During the past several decades, there has been a steady increase in the frequency of opportunistic fungal infections in immunocompromised individuals (Ascioglu et al. 2002). IPA is the most common fungal pulmonary infection in immunocompromised patients (Herbrecht et al. 2004). The main risk factors for aspergillosis are: hematopoietic stem cell and solid organ transplants, hematologic malignancy, AIDS and various pulmonary disorders (Patterson et al. 2000). The diagnosis of IPA is based on: clinical, radiological and mycological data. The clinical signs have low specificity. The most typical CT scan findings are: nodules with or without the halo or the air crescent sign (Herbrecht et al. 2004). High resolution MDCT angiography has been shown to be a feasible technique to depict directly vessel occlusion in the setting of suspected fungal infection, particularly for early diagnosis of angioinvasive pulmonary aspergillosis in immunocompromised hosts (Sonnet et al. 2005). The sensitivity of microscopy and culture of non-invasive collected samples is low. Galactomannan and nucleic acid detection in the serum or in the BAL fluid help to confirm the diagnosis (Herbrecht et al. 2004). However, the diagnosis of IPA can only be confirmed by histological determination of invasion of lung tissue by *Aspergillus* species or presence of positive cultures for *Aspergillus* in a sample obtained from a sterile site by an invasive procedure e.g. lung biopsy (Kristan et al. 2002; Ascioglu et al. 2002; Maertens et al. 2004). Open lung biopsy has very low morbidity and is recommended to establish the diagnosis of IPA. In a retrospective study performed in patients with malignant hematological disorders suspected of having IPA clinically and radiologically, only 53% of patients were proven to have IPA, the remaining 47% of patients were shown to have other etiologies e.g. organizing pneumonia, pulmonary hemorrhage or pneumonia due to other infections e.g. tuberculosis, CMV and candida (Kim et al. 2002). Recent studies have also shown that the lesions of IPA in neutropenic immunocompromised patients differ from those in non-neutropenic individuals in that they predominantly consist of angioinvasion and intraalveolar hemorrhage and that the innate host defences largely contribute to the histological patterns observed in IPA (Stergiopoulou et al. 2007). Optimal therapeutic stratigies include: prevention of contamination in at high risk patients, early initiation of antifungal therapy, surgical resection in patients having hemoptesis and lesions close to lage blood vessels and treatment of the underlying disease condition so as to restore a certain degree of immunity. The crude mortality is high and is strongly correlated not only with the type and the stage of the underlying disease but also with the extent of aspergillosis (Herbrecht et al. 2004; Patterson et al. 2000).

For many years, treatment of severe fungal infections had been limited to amphotericin-B and flucytosine. Fortunately, the past few years have brought remarkable advancements in antifungal pharmacotherapy as several new antifungals have been introduced (Battegay and Fluckiger, 2003). With the advent of the new triazoles e.g. voriconazole and the lipid formulations of amphotericin-B in addition to the echinocandins e.g. caspofungin, invasive aspergillosis has become treatable. The lipid formulations of amphoteircin-B have shown a clear advantage in reducing the side effects e.g. nephrotoxicity of amphotericin-B (Battegay and Fluckiger, 2003). Caspofungin has been shown to be as effective as and generally better tolerated than liposomal amphotericin B when given as empirical antifungal therapy in patients with prolonged neutropenia having persistent pyrexia (Walsh et al. 2004). It has demonstrated clinical efficacy when administered as first-line empirical therapy in patients with persistent febrile neutropenia, as salvage therapy in patients refractory to or intolerant of standard antifungal therapies and as combination therapy in difficult-to-treat patients refractory to or intolerant of standard therapies (Maertens, 2006). Voriconazole has become a primary therapeutic modality for invasive aspergillosis, despite the concerns about the development of drug-resistant fungi (Aperis and Mylonaki, 2006). Studies in patients with invasive aspergillosis have shown that voriconazole use led to better responses and improved survival and resulted in fewer severe adverse effects than the standard approach of initial therapy with amphotericin B (Herbrecht et al. 2002).

In case of intolerance to or failure of treatment with caspofungin or voriconazole, one of the new echinocandins e.g. micafungin or triazoles e.g. posaconazole can be used as an alternative salvage antifungal therapy (Walsh et al. 2007; Herbrecht et al. 2004).

Nocardia infections develop in patients with underlying lung pathology e.g. cystic fibrosis, bronchiectasis or chronic obstructive airway disease as well as in immunocompromised individuals e.g. those receiving corticosteroids and other immunosuppressive agents, organ transplant recipients and cancer patients specially those with lymphoreticular neoplasms. The most commonly involved organs are: the lungs, the central nervous system and the skin (Yildiz and Doganay, 2006; Matulionyte et al. 2004; Pifarre et al. 2001; Hui et al. 2003). However, disseminated infections are rather uncommon. The most frequently isolated species is *N. asteroides* (Matulionyte et al. 2004; Hui et al. 2003). Pulmonary nocardiosis is an important cause of opportunistic infections in immunocompromised hosts and the incidence of this infection is increasing (Yildiz and Doganay, 2006; Hui et al. 2003). It commonly presents as a chronic debilitating illness with radiological manifestations resembling pulmonary tuberculosis or lung cancer (Yildiz and Doganay, 2006; Matulionyte et al. 2004). In immunocompromised hosts, a fulminant disease resembling acute bacterial pneumonia may occasionally be encountered (Yildiz and Doganay, 2006). The median time between the onset of symptoms and the diagnosis of nocardia infection is about 30 days (Matulionyte et al. 2004). Clinically mild symptoms are common, but variable and nonspecific radiological changes including extensive nodular lung involvement that resemble cannonballs of metastatic cancer may be encountered (Pifarre et al. 2001; Hui et al. 2003; Tripodi et al. 2001). Recurrence of the infection and complications may also occur (Matulionyte et al. 2004). The diagnosis depends upon a high degree of suspicion so as to alert microbiologists and pathologists to employ special methods for identification of the organism (Yildiz and Doganay, 2006). Early diagnosis of pulmonary nocardiosis can be established upon culturing specimens of lung lesions obtained by BAL or fine-needle aspirate (Tripodi et al. 2001). Susceptibility studies and tests of antibiotic synergism should guide the therapy (Tripodi et al. 2001). The isolates are usually susceptible to: TMP/SMZ, carbapenems, amikacin, ceftriaxone, ciprofloxacin and tazobactam-piperacillin (Yildiz and Doganay, 2006; Matulionyte et al. 2004; Hui et al. 2003; Tripodi et al. 2001). Despite the development of drug resistance in some strains, the sulpha combinations e.g. TMP/SMZ are still the first line agents in the management of nocardiosis and carbapenems should be used as an alternative therapeutic modality in severely ill patients (Yildiz and Doganay, 2006; Matulionyte et al. 2004). A synergistic combination of a beta-lactam/beta-lactamase inhibitor with ciprofloxacin or amikacin followed by a short course of TMP/SMZ may result in eradication of nocardial disease and may reduce the need for long-term therapy (Tripodi et al. 2001). Delayed diagnosis, systemic infection and neurological involvement carry poor prognosis, while early recognition of the organism and prompt treatment have good outcome and may result in complete cures (Yildiz and Doganay, 2006; Matulionyte et al. 2004).

The patient presented was severely immunocompromised and had several predisposing factors for the invasive pulmonary infections encountered including: his advanced age; having multiple medical illnesses including bronchiectasis; having the T-cell lymphoma treated with various immunosuppressive drugs including corticosteroids; having a recent CMV infection and having moderately severe neutropenia prior to and during the latest hospitalization. We believe that the immunosuppressive therapy administered to this patient, particularly alemtuzumab, contributed not only to the development of both invasive lung infections, but also to the reactivation of CMV, particularly as he developed neutropenia and lymphopenia several months following this immununotherapy. He presented with severe bronchopneumonia clinically and diffuse nodular lung infiltration in addition to segmental consolidation radiologically. As he did not have appropriate antimicrobial cover initially, he developed the septic shock. However, the septic episode was successfully managed with meropenem, vancomycin in addition to the other supportive measures taken. Thereafter, the antimicrobial therapy was modified when nocardia and aspergillus were cultured from sputum and BAL fluid. Subsequently, the patient made an excellent clinical and radiological recovery although the treatment was rather prolonged.

## Conclusion

Even in septic immunocompromised hosts, slowly growing organisms and microorganisms that are difficult to culture or isolate should always be included in the differential diagnosis. Taking enough cultures and septic screens, making thorough and repeated physical assessment and requesting all the necessary investigations including high resolution CT scans are essential in the management of septic episodes and severe infections. At times, invasive diagnostic procedures such as bronchoscopy, BAL and even open lung biopsies may become essential to determine the causative microorganisms of certain pulmonary infections.
